# EXPLORING THE POWER OF COMBINATORIAL HEALTH TECHNOLOGIES TO SUPPORT SELF-MANAGEMENT OF COPD AMONG OLDER PEOPLE

**DOI:** 10.1093/geroni/igz038.1662

**Published:** 2019-11-08

**Authors:** Sandra Varey, Mandy Dixon, Alejandra Hernandez, Ceu Mateus, Tom Palmer, Christine Milligan

**Affiliations:** Lancaster University, Lancaster, United Kingdom

## Abstract

Ways to address the increasing healthcare needs of older people are a priority for the National Health Service (NHS) in England. The NHS England Test Bed programme was designed to trial new models of care that are supported by digital health technologies. This paper reports on findings from one Test Bed programme, the Lancashire and Cumbria Innovation Alliance (LCIA) – a partnership between NHS England, industry and Lancaster University, which ran from 2016 to 2018. A key aim of the LCIA Test Bed was to explore the extent to which supported self-care telehealth technology helped older people with long-term conditions to better self-manage their own care, promoting independence and enabling them to remain at home for longer. Each patient received a combination of health technologies over a six-month period. This paper presents results from the qualitative data that formed part of a large-scale mixed-methods evaluation. Specifically it draws on the analysis of 34 observational interviews with 17 participants with chronic obstructive pulmonary disease (COPD) to understand the role of these technologies in the self-management of their care. The data revealed that the majority of participants felt more confident about self-managing COPD as a result of their participation in the programme. These increases in confidence were the result of participants’ increased knowledge and skills in managing their COPD. The paper demonstrates how patients learned to better manage their respiratory condition, the impact of this learning on their daily lives and that of their family carers, and the implications for healthcare practice.

